# Dendrobine suppresses endoplasmic reticulum stress-induced apoptosis through upregulating microRNA miR-381-3p to decrease caspase-4

**DOI:** 10.1080/21655979.2021.1956672

**Published:** 2021-07-24

**Authors:** Jing Meng, Xiaoying Song, Guoliang Yan, Haihui Wang, Haitao Li, Danfei Lou

**Affiliations:** a Department of Geriatrics, Shanghai Municipal Hospital of Traditional Chinese Medicine, Shanghai University of Traditional Chinese Medicine, Shanghai, China; b Emergency Department, Shanghai Municipal Hospital of Traditional Chinese Medicine, Shanghai University of Traditional Chinese Medicine, China

**Keywords:** Dendrobine, miR-381-3p, caspase-4, endoplasmic reticulum stress, proliferation, apoptosis

## Abstract

Dendrobine has been reported to reduce blood lipid levels and apoptosis. The present study was designed to observe the effect of dendrobine in a model of ERS using vascular endothelial cells and to reveal the biological mechanisms and pathways responsible for the therapeutic effects of dendrobine on AS. Human umbilical vein endothelial cells (HUVECs) were pre-treated with various concentrations of dendrobine, followed by treatment with tunicamycin (TM) for the establishment of the cell models of ERS. The proliferation and apoptosis of HUVECs were detected by bromodeoxyuridine staining and flow cytometry, respectively. The target binding association was verified through dual luciferase reporter assay. It was found that TM treatment resulted in a low expression of miR-381-3p. Dendrobine treatment not only promoted the proliferation, but also inhibited the apoptosis of HUVECs induced by TM. The reduced expression of 78-kDa glucose-regulated protein, inositol-requiring enzyme 1, caspase-4, C/EBP homologous protein and caspase-3 was also observed following treatment with dendrobine. Dendrobine reduced the apoptosis of endothelial cells in the model of ERS by increasing miR-381-3p expression, and partially restored the cell proliferation level. This effect was significantly reduced after the expression of miR-381-3p was blocked. On the whole, the present study demonstrated that dendrobine upregulated miR-381-3p expression to inhibit apoptosis induced by ERS in HUVECs and this process was found to be mediated by caspase-4. The findings of the present study may provide new insight into the causes of endothelial cell apoptosis during AS and reveal the potent therapeutic effects of dendrobine in AS.

## GRAPHICAL ABSTRACT

**Figure:**
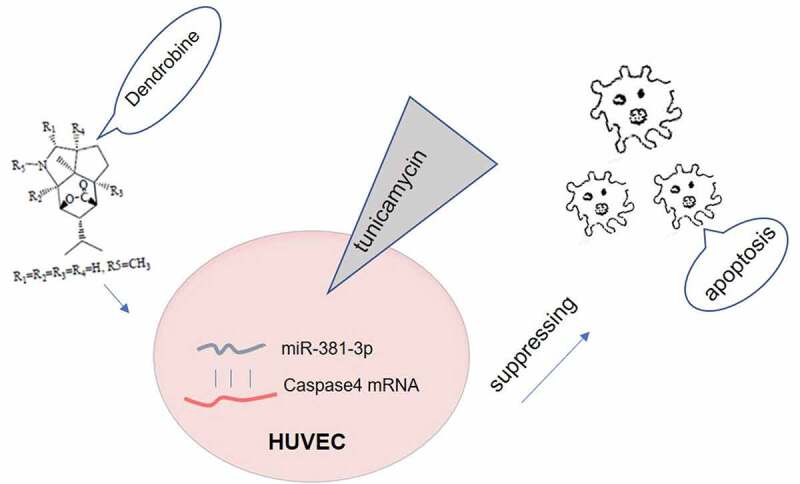

## Introduction

Atherosclerosis (AS) represents the main pathological basis of the majority of cardiovascular diseases, which are one of the most challenging problems in current medicine [1]. AS is a complex process involving a variety of metabolic and signaling pathways. In recent years, increasing evidence has indicated that endoplasmic reticulum stress (ERS) is closely associated with AS [2]. The apoptosis of vascular endothelial cells is a key mechanism of AS. ERS and the subsequent induction of apoptosis play an important role in the early stages of AS [3,4].

Dendrobine is widely distributed in tropical and subtropical regions of Europe, Asia and Oceania. Dendrobine is mainly derived from *Dendrobium nobile*, and its bioactivity is similar to that of picrotoxin. Dendrobium was determined to be the first picrotoxane-type alkaloid detected by chemical and spectral methods [5,6]. MicroRNAs (miRNAs/miRs) are a class of endogenous small-molecule non-coding single-stranded RNAs, which are composed of ~22 single nucleotides. miRNAs interact with targets through the complementary base pairing of the seed region of miRNAs with the 3ʹ-untranslated region (UTR) of their homologous target mRNAs [7–9]. One of the mechanisms through which miRNAs affect endothelial dysfunction is through their effects on endothelial apoptosis [10,11]. Endothelial cell injury can lead to the destruction of endothelial completion and barrier function, and can promote lipid deposition, leading to the development of (AS) [12]. The coding gene of human miR-381 (hsa-miR-381) is located on the long arm of chromosome 14 and belongs to the miR-154 gene family, which is involved in the apoptosis and proliferation of endothelial cells [13]. A previous study demonstrated that miR-381-3p was significantly downregulated in the AS group compared with the normal group [14]. Another study reported that there was a direct negative association between the miR-381-3p levels and the sensitivity to apoptosis; the ectopic expression of miR-381-3p exerted an inhibitory effect on the activation of caspase-3 [15]. Dendrobine has been shown to have the ability to lower glucose and lipid levels, exert antitumor effects, reduce inflammation and apoptosis, and to attenuate cell senescence [16,17]. However, the underlying molecular mechanisms remain unclear.

We predicted that miR-381-3p could be involved in the effects of dendrobine on AS. The aim of the present study was to elucidate the molecular mechanisms through which dendrobine inhibits ERS and protects human umbilical vein endothelial cells (HUVECs) from apoptosis, and to determine the role of miR-381-3p in this process.

## Materials and methods

*Cells and cell culture*. The HUVEC cell line (ATCC® CRL-1730™ American Type Culture Collection) was cultured in DMEM containing 10% FBS with 100 mg/l penicillin/streptomycin in 37°C with 5% CO_2_. The endothelial cells were cultured in a 6-well plate and the medium was changed every two days. When the cell confluence reached 70–80%, the cells in a proliferative state were cultured with DMEM containing 1% FBS for 24 h to synchronize cell proliferation. The HUVEC model of ERS was established by the use of tunicamycin (TM, 2 μM) for 12 h. HUVECs in the tauroursodeoxycholic acid (TUDCA) or dendrobium groups were treated with TUDCA or dendrobium for 12 h prior to TM treatment, followed by the addition of TM for 12 h. Dendrobium was purchased from Shanghai yuanye Bio-Technology Co., Ltd (HPLC≥98%, Shanghai, China). TUDCA has been investigated to be as an endoplasmic reticulum stress inhibitor [18,19], which was used to be as a positive control group in this study. TM was used to establish an ERs model, As descripted previously [20].

*Cell Counting Kit (CCK)-8 assay*. HUVECs (2x10^4^ cells) were seeded into a 96-well plate and subjected to various treatment conditions. After 24 h, 10 µl CCK-8 solution (Thermo Fisher Scientific, Inc.) were added to each well. The absorbance at 450 nm was detected after 4 h using a microplate reader.

*Bromodeoxyuridine (BrdU) staining*. HUVECs (1.5x10^5^/ml) were seeded into cell culture dishes. Following experimental intervention, BrdU (Thermo Fisher SCIENTIFIC. Inc) was added to the cell culture dishes followed by incubation for 40 min at 37°C. The culture medium was then discarded and the cells were fixed with 100% methanol for 10 min. Subsequently, the cells were incubated overnight at 4°C with AlexaFluor® 647 Anti-BrdU antibody (ab220075, Abcam), followed by the labeling of nuclear DNA with DAPI.

*TUNEL assay*. The apoptotic levels of HUVECs were assessed using a TUNEL assay kit following the manufacturer’s protocol (Roche Diagnostics). The cells were washed with PBS and then fixed with 4% paraformaldehyde. PBS containing 0.1% Triton X-100 was then added followed by incubation for 2 min. A total of 50 μl TUNEL solution was then added to samples followed by incubation at 37°C for 60 min in the dark. The apoptotic cells of 5 random fields were observed under a fluorescence microscope (OLYMPUS) after fluorescence quenching solution was used to seal the plates.

*Western blot analysis*. After each group was treated, total protein was extracted using RIPA lysis solution (Sigma Aldrich) and the protein concentration was determined by the Bradford method. A total of 50 µg protein sample was loaded into each well and subjected to SDS-PAGE gel electrophoresis to separate target proteins. Subsequently, proteins were transferred into PVDF membrane. The protein bands were then blocked in 5% skimmed milk powder at room temperature for 2 h and then incubated with primary antibodies (GRP78, cat.no ab108615, dilution of 1:1000. IRE1, cat.no ab124945, dilution of 1:1000. Caspase4, cat.no ab25898, dilution of 1:1000. Chop, cat.no ab11419, dilution of 1:500. Caspase3, cat.no ab32351, dilution of 1:5000. GAPDH, ab8245, dilution of 1:10,000, Abcam) at 4°C overnight, followed by incubation with secondary antibodies (goat anti-mouse IgG, cat. no. ab216772; Goat Anti-Rabbit IgG, cat. no. ab9705, 1:10,000.). After ECL solution (Pierce™ ECL Western Blotting Substrate, ThermoFisher Scientific. Inc) was used to develop color, the gray value was analyzed using ImageJ 1.42 r (National Institutes of Health).

*Reverse transcription-quantitative PCR (RT-qPCR)*. Total RNA and miRNA of the cells to be tested were extracted using the total RNA extraction kit and miRNEasy Mini kit (QIAGEN, Germany), respectively. The concentration and purity of RNA were determined by ultraviolet spectrophotometry. Using RNA as a template, cDNA was synthesized by reverse transcription according to the protocol provided with the corresponding reverse transcription kit (Takara Bio, Inc.). The cDNA template was then used for fluorescence qPCR detection using the miRNA qRT-PCR SYBR® kit (Clontech, USA). For the detection of caspase4 mRNA, PCR quantification was performed using SYBR Green qPCR Master Mix (MedChemExpress). The PCR cycle condition contains preheat for 5 min at 94°C, 35 cycles of degeneration at 94°C for 10s, annealing at 55°C for 10s and extension at 72°C for 30s and at 72°C for 10 min. U6 or GAPDH was used as the internal reference and the relative levels of miR-381-3p or caspase4 mRNA were calculated using the 2^−ΔΔCq^ method [21]. The primers used in this study are as following: miR-381-3p: Forward, 5ʹ-TACTTAAAGCGAGGTTGCCCTT-3ʹ; Reverse, 5ʹ-GGCAAGCTCTCTGTGAGTA-3ʹ. Caspase4: Forward, 5ʹ – CAAGAGAAGCAACGTATGGCA-3ʹ; Reverse, 5ʹ – AGGCAGATGGTCAAACTCTGTA-3ʹ. U6 Forward, 5ʹ-CTCGCTTCGGCAGCACA-3ʹ; Reverse, 5ʹAACGCTTCACGAATTTGCGT-3ʹ. GAPDH, Forward, 5ʹ – TGACGTGCCGCCTGGAGAAC −3ʹ; Reverse, 5ʹ – CCGGCATCGAAGGTGGAAGAG −3ʹ.

*Dual-luciferase reporter assay*. The online target gene prediction database, starBase, was used to predict the target genes of miR-381-3p. The plasmids containing the binding site (wt-caspase-4) and mutation sequence (mut-caspase-4) were designed and synthesized according to the predicted binding sites mentioned above from gas-phase electrophoretic molecular mobility analysis. Wt-caspase-4 or mut-caspase-4 was cloned into a luciferase reporter vector psiCHECK2 (Ke Lei Biological Technology Co., Ltd. Shanghai, China) to construct a fluorescence reporter plasmid. The cells were subjected to the cotransfection of miR-NC and miR-381-3p. After 48 h at 37°C, the luciferase activity was then detected. The specific operation method was performed according to the requirements of the dual luciferase reporter assay kit (Yeasen, Shanghai, China), and the luciferase activity of firefly and renal fluorescein were recorded.

*miRNA transfection*. miR-381-3p mimic (50 nM), inhibitor (50 nM) or negative control were synthesized and purchased from Shanghai GenePharma Co., Ltd. These miRNA reagents were transfected into HUVECs using Lipofectamine® 3000 (Invitrogen; Thermo Fisher Scientific, Inc.) at 37°C for 24 h according to manufacturer’s protocol and subsequently used in further experiments. Untransfected cells were used to be as control groups. The sequences of miR reagent are as following: miR-381-3p mimic 5ʹ-UAUACAAGGGCAAGCUCUCUGU-3ʹ, miR mimic-NC, 5ʹ-UAUCCUAUGGCUAGGAAGUACC-3ʹ. miR-381-3p inhibitor, 5ʹAUAUGUUCCCGUUCGAGAGACA-3ʹ, miR inhibitor-NC, 5ʹ – GGACAUGUGUAAUCCAUCUGCA-3ʹ

*Statistical analysis*. All the tests were repeated at least three times, and GraphPad Prism 7. 0 statistical software (GraphPad Software, Inc.) was used to analyze the data and prepare statistical graphs. The experimental data were presented as mean ± standard deviation (SD). One-way ANOVA was used for comparisons between multiple groups followed by Tukey’s post hoc test. *P < *0.05 was considered to indicate a statistically significant difference.

## Results

**Figure 1.**
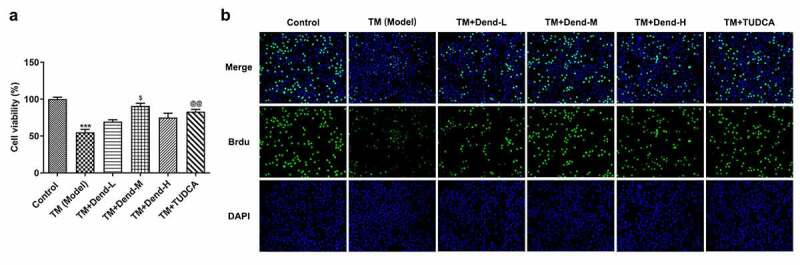
Effects of dendrobine on HUVEC proliferation. (a) Results of Cell Counting Kit-8 assay. (b) Evaluation of HUVEC proliferation through BrdU staining. Dend-L: the low concentration of dendrobine, 1 µM; Dend-M: the medium concentration of dendrobine, 5 µM; Dend-H: the high concentration of dendrobine, 10 µM. HUVEC, human umbilical vein endothelial cell; BrdU, bromodeoxyuridine. ***P < 0.001 Vs Control, ^$^*P < *0.05 Vs TM+Dend-L, ^@@^*P < *0.01 VS TM (Model)

**Figure 2.**
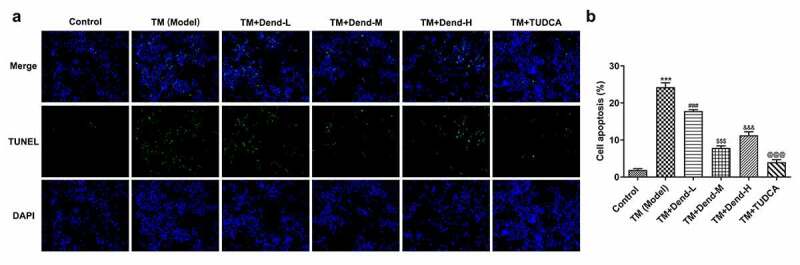
Effects of dendrobine on apoptosis. (a and b) Results of TUNEL staining for apoptosis. ***P < 0.001 Vs Control, ###*P < *0.001 Vs TM (Model), ^$$$^*P < *0.001 Vs TM+Dend-L, ^&&&^*P < *0.001 Vs TM+Dend-M, ^@@@^*P < *0.01 VS TM (Model)

*Dendrobine attenuates the proliferation-suppressive and apoptosis-inducing effects of TM on HUVECs*. In order to examine the effects of dendrobine on ERS in HUVECs, the cells were treated with various concentrations of dendrobine, followed by treatment with TM. We used TUDCA to suppress ERs, which has been investigated to be as an endoplasmic reticulum stress inhibitor [18,19]. As shown in Figures 1 and 2, increased cell viability was observed following treatment with the medium concentration of dendrobine, an effect which was also appeared in the TUDCA-treated cells (*P < *0.05, Figure 1a). When BrdU staining was performed to examine the proliferation of HUVECs, an evident increase in green fluorescence was observed only with the medium concentration of dendrobine, an effect which was also observed with TUDCA treatment (Figure 1b). By contrast, the results of TUNEL assay for the analysis of the cell apoptosis levels revealed that the fluorescence intensity in response to treatment with the medium concentration of dendrobine was significantly decreased when compared with the model group (*P < *0.001, Figure 2b). Similar effects were also observed in the positive control group treated with TUDCA. These results indicated that the alteration in proliferation and apoptosis induced by treatment with the medium concentration of dendrobine was similar to the effect induced by the ERS inhibitor. Thus, dendrobine plays a protective role in HUVECs subjected to ERS.

**Figure 3.**
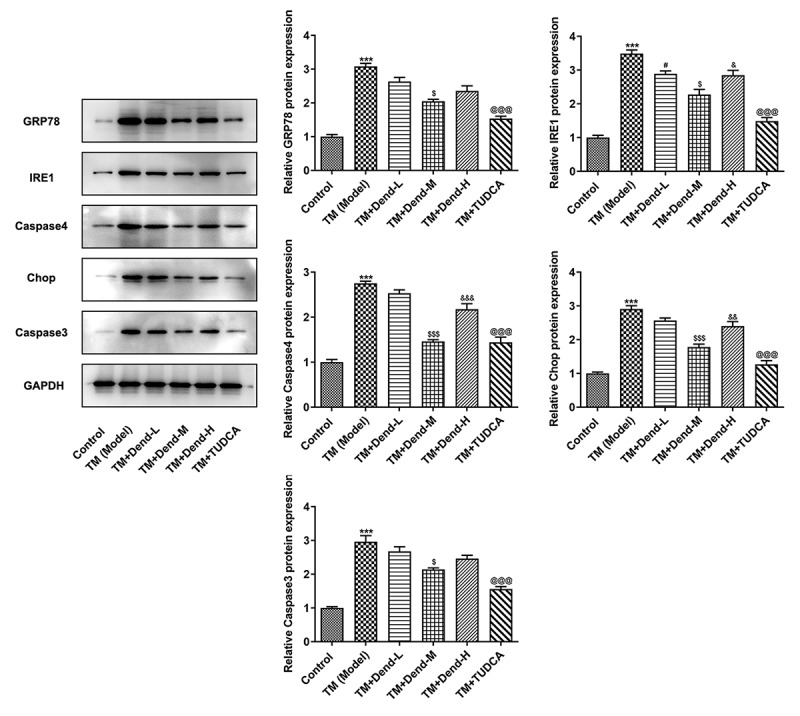
Assessment of endoplasmic reticulum stress-related proteins levels following treatment with dendrobine. Results of western blot analysis of GRP78, Ire1, caspase-4, CHOP and caspase3 expression. GRP78, 78-kDa glucose-regulated protein; Ire1, inositol-requiring enzyme 1; CHOP, C/EBP homologous protein. ***P < 0.001 Vs Control, #*P < *0.05 Vs TM (Model), ^$^*P < *0.05, ^$$$^*P < *0.001 or Vs TM+Dend-L, ^&^*P < *0.05, ^&&^*P < *0.01 or ^&&&^*P < *0.001 Vs TM+Dend-M, ^@@@^*P < *0.01 VS TM (Model)

**Figure 4.**
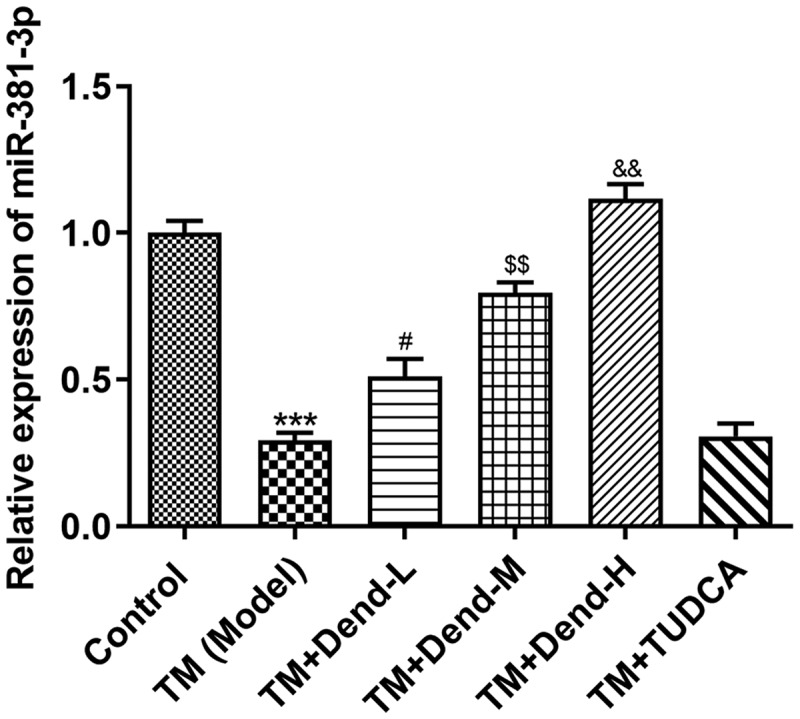
Assessment of miR-381-3p expression following treatment with dendrobine. Results of reverse transcription-quantitative PCR analysis for miR-381-3p expression. miR, microRNA. ***P < 0.001 Vs Control, #*P < *0.05 Vs TM (Model), ^$$^*P < *0.01 or Vs TM+Dend-L, ^&&^*P < *0.01 Vs TM+Dend-M, ^@@@^*P < *0.01 VS TM (Model)

*Alteration of ERS-related protein expression through dendrobine treatment*. To examine the molecular mechanisms involved in the effects of dendrobine, focus was mainly paid to the ERS pathway. Western blot analysis of the expression of 78-kDa glucose-regulated protein (GRP78), inositol-requiring enzyme 1 (Ire1), caspase-4, C/EBP homologous protein (CHOP) and caspase-3 demonstrated that the expression of these proteins was markedly reduced following dendrobine treatment at the medium concentration (*P < *0.05) when compared with the model group; the expression of these proteins was not markedly altered following treatment with dendrobine at the low concentration, with the exception of Ire1 (Figure 3). Under conditions of ERS, the HUVECs expressed low levels of miR-381-3p; however, dendrobine pre-treatment significantly recovered these levels in a concentration-dependent manner (*P < *0.05). In addition, no significant changes in miR-381-3p expression were observed following treatment with TUDCA (Figure 4), suggesting that there was no direct association between TUDCA and miR-381-3p levels; thus, TUDCA may regulate ERS through other pathways.

**Figure 5.**
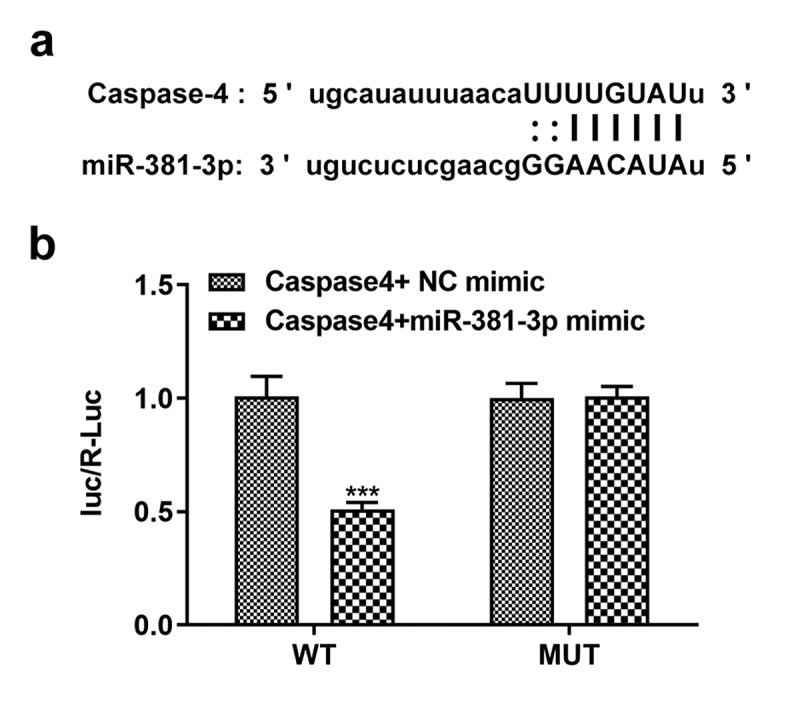
miR-381-3p binds to the 3ʹ-untranslated regions of caspase-4. (a) Binding sites predicted by starBase. (b) Results of dual-luciferase reporter assay. miR, micoRNA. ***P < 0.001 Vs WT+ Caspase4+ NC mimic

**Figure 6.**
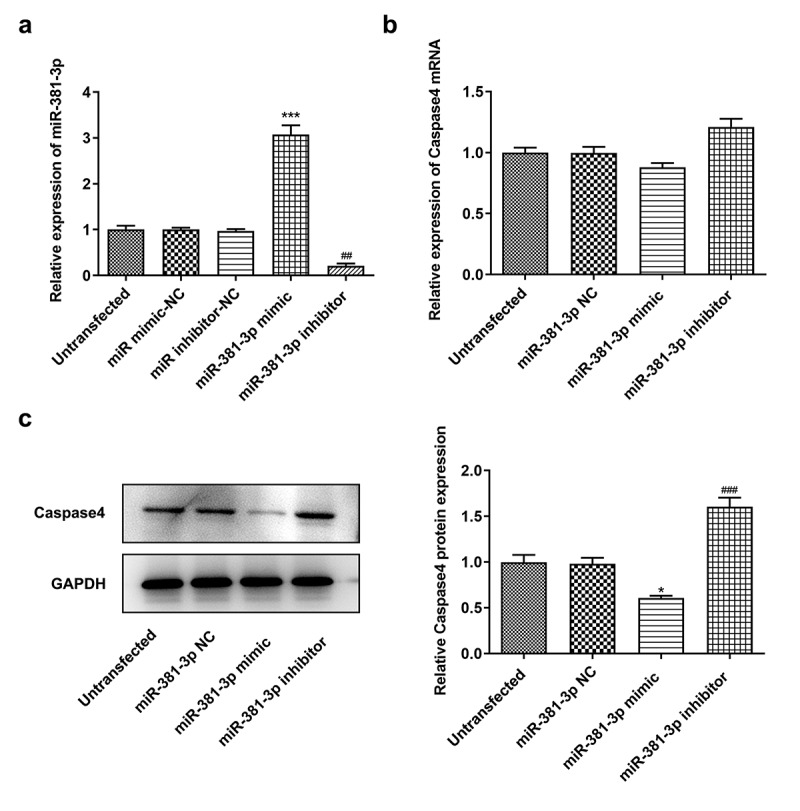
miR-381-3p affected the protein levels of caspase-4. (a) Reverse transcription-quantitative PCR analysis of miR-381-3p levels. (b) mRNA levels of caspase-4 following miR-381-3p mimic or inhibitor transfection. (c) Protein levels of caspase-4 following miR-381-3p mimic or inhibitor transfection. miR, microRNA. ***P < 0.001 Vs miR-381-3p NC, ^###^ *P < *0.001 Vs miR-381-3p mimic

**Figure 7.**
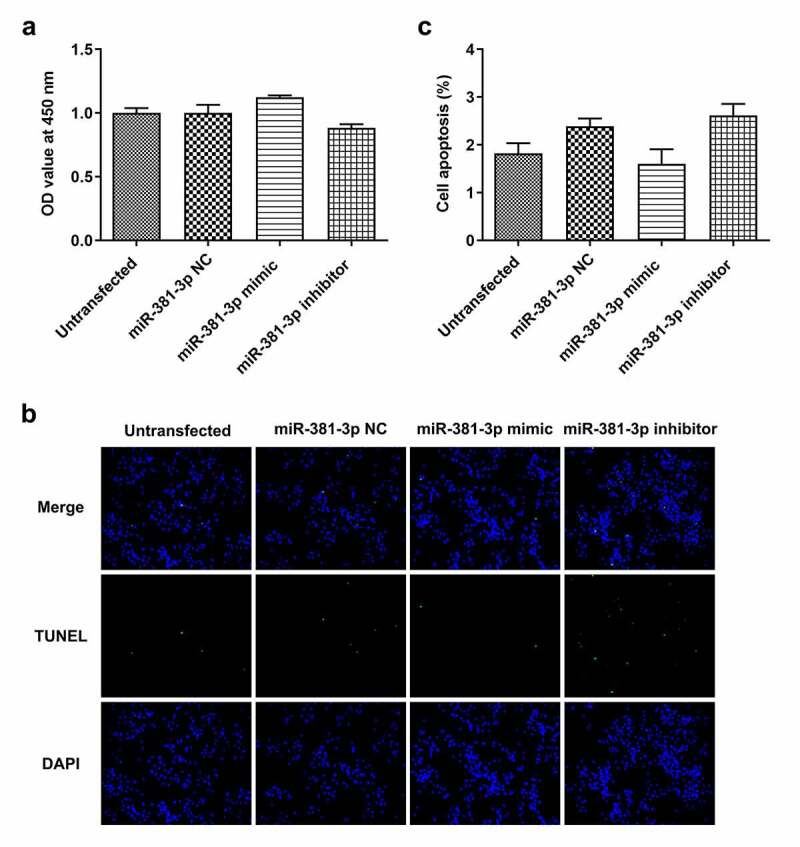
Effects of miR-381-3p mimic or inhibitor on cell proliferation and apoptosis. (a) Results of Cell Counting Kit-8 assay for cell proliferation following miR-381-3p mimic or inhibitor transfection. (b and c) Results of TUNEL staining for cell apoptosis following miR-381-3p mimic or inhibitor transfection. miR, microRNA

*No changes in cell proliferation and apoptosis are observed in HUVECs not subjected to ERS*. To verify the target association of miR-381-3p and caspase-4, a dual-luciferase reporter assay was performed to determine whether miR-381-3p binds to the 3ʹUTR of caspase-4 mRNA. Notably, the luciferase activity was reduced (*P < *0.001) when WT caspase-4 and miR-381-3p mimic were co-transfected into the cells with incubation for 48 h (Figure 5a, b). To further investigate the role of miR-381-3p, the effects of transfection with miR-381-3p mimic or inhibitor were first examined. The results of RT-qPCR revealed that miR-381-3p mimic or inhibitor significantly altered the levels of miR-381-3p (Figure 6a). However, in HUVECs not challenged with TM, miR-381-3p did not affect the mRNA levels of caspase-4 (Figure 6b), whereas it affected the protein levels of caspase-4 (*P < *0.01, Figure 6c). In addition, no significant changes were observed in the proliferation and apoptosis of HUVECs transfected with miR-381-3p mimic or inhibitor (Figure 7a-c). These results indicated that miR-381-3p did not affect cell proliferation or apoptosis when the HUVECs expressed basal caspase-4 levels without ERS induction.

**Figure 8.**
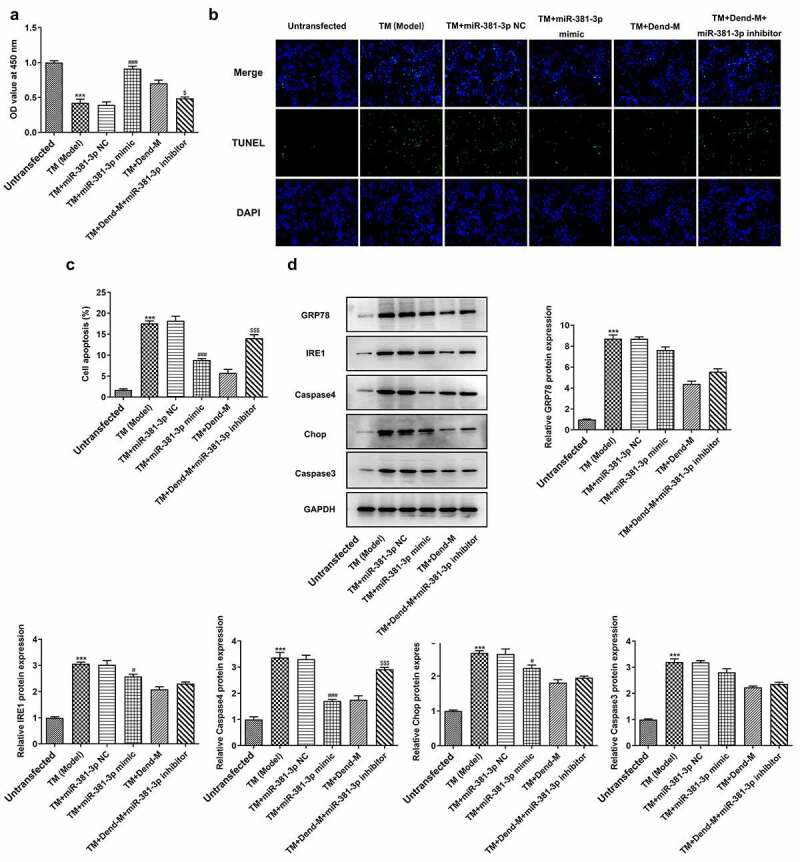
Effects of miR-381-3p mimic or inhibitor on cell proliferation and apoptosis following TM induction. (a) Results of Cell Counting Kit-8 assay. (b and c) Evaluation of apoptosis through TUNEL assay. (d) Detection of ERS-related proteins levels through western blot analysis. TM, tunicamycin; ERS, endoplasmic reticulum stress; miR, microRNA. ***P < 0.001 Vs Control, ^###^ *P < *0.001 Vs TM+miR-381-3p NC, <0.05, ^$$$^*P < *0.001 Vs TM+Dend-M

*miR-381-3 mediates the effects of dendrobine on HUVECs subjected to ERS*. To determine whether miR-381-3p inhibitor alters the effects of dendrobine on HUVECs challenged with TM, the TM-induced cells were treated with dendrobine and miR-381-3p inhibitor. The aforementioned cellular processes, including proliferation and apoptosis, were markedly altered in the HUVECs treated with TM following overexpression of miR-381-3p. Of note, miR-381-3p inhibitor significantly reversed the effects of dendrobine on HUVECs treated with TM (*P < *0.05, Figure 8a-c). Western blot analysis was then performed to examine the expression of ERS-related proteins. A significant decrease in caspase-4 expression was observed in the miR-381-3p-overexpressing HUVECs subjected to ERS; however, transfection with miR-381-3p inhibitor reversed the effects of dendrobine on these levels (*P < *0.05, Figure 8d). Furthermore, these effects of miR-381-3p were not observed in other proteins. Therefore, in addition to the involvement of caspase-4-related apoptosis mediated by miR-381-3p in the effects of dendrobine, other ERS pathways are also involved in the mechanisms of action of dendrobine.

## Discussion

The alterations of endothelial cells lead to different phases of AS. The influence of ER stress on endothelial disfunction has been revealed in recent studies [22,23]. The proliferation and apoptosis of HUVECs play crucial roles in the pathogenesis of AS [24,25]. Normal level of cell apoptosis is beneficial for maintaining physiological homeostasis. The apoptosis of endothelial cell is associated with the development of AS [26]. In addition to apoptosis, pyroptosis or ferroptosis of endothelial cells was also reported to be implicated in pathological mechanisms of atherosclerosis [27,28]. Emerging evidences have revealed that dendrobine has the effects of lowering glucose. In the present study, TM was used to induce ERS in HUVECs, which suppressed cell proliferation and induced apoptosis, together with the induction of ERS-related protein expression. TUCDA is an ERs inhibitor, which was used to as positive control group. The appropriate concentration of dendrobine enhanced the proliferation and promoted the apoptosis of HUVECs exposed to TM, accompanied by a marked decrease in the levels of GRP78, Ire1, CHOP and caspase-3. Dendrobine was reported to reduce inflammation in gestational diabetes mellitus (GDM) and produces a protective effect on ameliorating the symptoms of GDM and isoniazid – and rifampicin-induced liver injury [29,30]. In addition, it has a potential anti-tumor effect on non-small cell lung cancer [16]. Therefore, we predict that dendrobine exerts different effects on cancer and non-cancer possibly due to the differentiation of cells environment of different types. Persistent or excessive ER stress would induce apoptosis transcription by transcription factor-dependent, and caspase 12-dependent pathways. GRP78/IRE1/CHOP axis constitutes transcription factor-dependent pathway [31,32]. Caspase-4, an alternative to caspase-12 in humans, is a downstream apoptotic signaling protein of ERS, and is a specific target of free Ire1, which is mainly localized in the outer membrane of the ER [33,34]. CHOP is another ERS apoptotic signaling protein that is not directly regulated by Ire1 [23,35]. In the present study, under non-stress conditions, miR-381-3p mimic or inhibitor did not have significant effects on the levels of caspase4 mRNA, but markedly affect the protein levels of caspase4, which could result from low basal levels of caspase4. After ERs induction, HUVEC cells displayed dramatically increased mRNA and protein levels of caspase4 while miR-381-3p mimic was able to markedly reduce the mRNA and protein levels of caspase-4. Furthermore, its inhibitor reversed the effects of dendrobine on caspase-4 levels in HUVECs challenged with TM.

Although significant inhibitory effects of dendrobine on GRP78, Ire1, caspase-3 and CHOP expression were observed, no significant changes in the expression levels of these proteins were observed following miR-318-3p overexpression. This suggested that, apart from the promoting role of dendrobium on miR-381-3p expression to suppress caspase-4-mediated endothelial cell apoptosis, dendrobine may also promote proliferation and inhibit apoptosis via other mechanisms related to other ERS pathways. To conclude, dendrobine at the medium concentration tested, helped the cells to combat the ERS caused by TM by inducing the upregulation of miR-381-3p expression, thereby reducing caspase-4 levels in an attempt to decrease ERS-mediated apoptosis.

## Conclusion

The present study demonstrated that dendrobine reduced ERS-induced apoptosis by upregulating miR-381-3p to decrease caspase-4 expression in HUVECs. The findings of the present study uncovered the effects and mechanisms of action of dendrobine in HUVECs challenged with TM. The data presented herein may thus provide additional insight into the role of ERS in the pathology of AS.

## Data Availability

The datasets used and/or analyzed during the current study are available from the corresponding author on reasonable request.

## Highlights

1. Dendrobine plays a protective role against human umbilical vein endothelial cells proliferation inhibition and apoptosis promotion induced by ERS.2. The effects of dendrobine on preventing ERS-induced cell injury are related to miR-381-3p.

3. Dendrobine decreases cell apoptosis caused by ERs is partly through decreasing caspase4, thereby blocking ERs-induced apoptosis.

4. Dendrobine could be a potential drug for the therapy of AS.

## References

[1] Benjamin EJ, Virani SS, Callaway CW, et al. Heart disease and stroke statistics-2018 update: a report from the american heart association. Circulation. 2018;137:e67–e492. 29386200 10.1161/CIR.0000000000000558

[2] Ivanova EA, Orekhov AN. The role of endoplasmic reticulum stress and unfolded protein response in atherosclerosis. Int J Mol Sci. 2016;17(2):193. 10.3390/ijms17020193 PMC4783927 26840309

[3] Hetz C. The unfolded protein response: controlling cell fate decisions under ER stress and beyond. Nat Rev Mol Cell Biol. 2012;13(2):89–102. 22251901 10.1038/nrm3270

[4] Di Pasquale E, Condorelli G. Endoplasmic reticulum stress at the crossroads of progeria and atherosclerosis. EMBO Mol Med. 2019;11(4). DOI:10.15252/emmm.201910360 PMC6460347 30902910

[5] Li R, Liu T, Liu M, et al. Anti-influenza A virus activity of dendrobine and its mechanism of action. J Agric Food Chem. 2017;65(18):3665–3674. 28417634 10.1021/acs.jafc.7b00276

[6] Liu GY, Tan L, Cheng L, et al. Dendrobine-type alkaloids and bibenzyl derivatives from Dendrobium findlayanum. Fitoterapia. 2020;142:104497. 32058054 10.1016/j.fitote.2020.104497

[7] Agrawal N, Dasaradhi PV, Mohmmed A, et al. RNA interference: biology, mechanism, and applications, microbiology and molecular biology reviews: Microbiol Mol Biol Rev. 2003;67:657–685. 10.1128/MMBR.67.4.657-685.2003 PMC309050 14665679

[8] He S, Zhang W, Li X, et al. Oral squamous cell carcinoma (OSCC)-derived exosomal MiR-221 targets and regulates phosphoinositide-3-kinase regulatory subunit 1 (PIK3R1) to promote human umbilical vein endothelial cells migration and tube formation. Bioengineered. 2021;12(1):2164–2174. 34098850 10.1080/21655979.2021.1932222 PMC8806445

[9] Zhuang X, Gao F, Shi L, et al. MicroRNA-146b-3p regulates the dysfunction of vascular smooth muscle cells via repressing phosphoinositide-3 kinase catalytic subunit gamma. Bioengineered. 2021;12(1):2627–2638. 34115567 10.1080/21655979.2021.1937904 PMC8806462

[10] Zhang Y, Liu X, Bai X, et al. Melatonin prevents endothelial cell pyroptosis via regulation of long noncoding RNA MEG3/miR-223/NLRP3 axis. J Pineal Res. 2018;64(2):e12449. 10.1111/jpi.12449 29024030

[11] Kumar S, Williams D, Sur S, et al. Role of flow-sensitive microRNAs and long noncoding RNAs in vascular dysfunction and atherosclerosis. Vascul Pharmacol. 2019;114:76–92. 30300747 10.1016/j.vph.2018.10.001 PMC6905428

[12] Wei DH, Jia XY, Liu YH, et al. Cathepsin L stimulates autophagy and inhibits apoptosis of ox-LDL-induced endothelial cells: potential role in atherosclerosis. Int J Mol Med. 2013;31(2):400–406. 23229094 10.3892/ijmm.2012.1201

[13] Guo Q, Yin X, Gao J, et al. MiR-381-3p redistributes between cytosol and mitochondria and aggravates endothelial cell injury induced by reactive oxygen species. Tissue Cell. 2020;67:101451. 33137708 10.1016/j.tice.2020.101451

[14] Song K, Li L, Sun G, et al. MicroRNA-381 regulates the occurrence and immune responses of coronary atherosclerosis via cyclooxygenase-2. Exp Ther Med. 2018;15:4557–4563. 29725388 10.3892/etm.2018.5947 PMC5920484

[15] Zhao C, Zhou Y, Ran Q, et al. MicroRNA-381-3p functions as a dual suppressor of apoptosis and necroptosis and promotes proliferation of renal cancer cells. Front Cell Dev Biol. 2020;8:290. 32411707 10.3389/fcell.2020.00290 PMC7198711

[16] Song TH, Chen XX, Lee CK, et al. Dendrobine targeting JNK stress signaling to sensitize chemotoxicity of cisplatin against non-small cell lung cancer cells in vitro and in vivo. Phytomedicine. 2019;53:18–27. 30668397 10.1016/j.phymed.2018.06.018

[17] Feng Y, Jia B, Feng Q, et al. Dendrobine attenuates gestational diabetes mellitus in mice by inhibiting Th17 cells, Basic & clinical pharmacology & toxicology. 2021;128(3):379–385. 10.1111/bcpt.13524 33119198

[18] Yue J, Wei YJ, Yang XL, et al. NLRP3 inflammasome and endoplasmic reticulum stress in the epileptogenic zone in temporal lobe epilepsy: molecular insights into their interdependence. Neuropathol Appl Neurobiol. 2020;46(7):770–785. 32311777 10.1111/nan.12621

[19] Li P, Fu D, Sheng Q, et al. TUDCA attenuates intestinal injury and inhibits endoplasmic reticulum stress-mediated intestinal cell apoptosis in necrotizing enterocolitis. Int Immunopharmacol. 2019;74:105665. 31254957 10.1016/j.intimp.2019.05.050

[20] Gundamaraju R, Vemuri R, Chong WC, et al. Interplay between endoplasmic reticular stress and survivin in colonic epithelial cells. Cells. 2018;7(10):171. 10.3390/cells7100171 PMC6210275 30326660

[21] Livak KJ, Schmittgen TD. Analysis of relative gene expression data using real-time quantitative PCR and the 2(-delta delta C(T)) method. Methods. 2001;25(4):402–408. 11846609 10.1006/meth.2001.1262

[22] Luchetti F, Crinelli R, Nasoni MG, et al. Secosterol-B affects endoplasmic reticulum structure in endothelial cells. J Steroid Biochem Mol Biol. 2019;190:234–241. 30991093 10.1016/j.jsbmb.2019.04.014

[23] Yi S, Chen K, Zhang L, et al. Endoplasmic reticulum stress is involved in stress-induced hypothalamic neuronal injury in rats via the PERK-ATF4-CHOP and IRE1-ASK1-JNK pathways. Front Cell Neurosci. 2019;13:190. 31130849 10.3389/fncel.2019.00190 PMC6509942

[24] Watanabe T, Sato K. Roles of the kisspeptin/GPR54 system in pathomechanisms of atherosclerosis, nutrition, metabolism, and cardiovascular diseases:Nutr Metab Cardiovasc Dis. 2020;30(6):889–895. 10.1016/j.numecd.2020.02.017 32409274

[25] Su T, Wang YB, Han D, et al. Multimodality imaging of angiogenesis in a rabbit atherosclerotic model by GEBP11 peptide targeted nanoparticles. Theranostics. 2017;7(19):4791–4804. 29187904 10.7150/thno.20767 PMC5706100

[26] Paone S, Baxter AA, Hulett MD, et al. Endothelial cell apoptosis and the role of endothelial cell-derived extracellular vesicles in the progression of atherosclerosis. Cell Mol Life Sci. 2019;76(6):1093–1106. 30569278 10.1007/s00018-018-2983-9 PMC11105274

[27] Wu X, Zhang H, Qi W, et al. Nicotine promotes atherosclerosis via ROS-NLRP3-mediated endothelial cell pyroptosis. Cell Death Dis. 2018;9(2):171. 29416034 10.1038/s41419-017-0257-3 PMC5833729

[28] Bai T, Li M, Liu Y, et al. Inhibition of ferroptosis alleviates atherosclerosis through attenuating lipid peroxidation and endothelial dysfunction in mouse aortic endothelial cell. Free Radic Biol Med. 2020;160:92–102. 32768568 10.1016/j.freeradbiomed.2020.07.026

[29] Feng Y, Jia B, Feng Q, et al. Dendrobine attenuates gestational diabetes mellitus in mice by inhibiting Th17 cells. Basic Clin Pharmacol Toxicol. 2021;128(3):379–385. 33119198 10.1111/bcpt.13524

[30] Garcia-Carracedo D, Cai Y, Qiu W, et al. PIK3CA and p53 mutations promote 4NQO-initated head and neck tumor progression and metastasis in mice, molecular cancer research: Mol Cancer Res. 2020;18(6):822–834. 10.1158/1541-7786.MCR-19-0549 PMC7272268 32152233

[31] Kamarehei M, Kabudanian Ardestani S, Firouzi M, et al. Increased expression of endoplasmic reticulum stress-related caspase-12 and CHOP in the hippocampus of EAE mice. Brain Res Bull. 2019;147:174–182. 30738137 10.1016/j.brainresbull.2019.01.020

[32] Zuo S, Kong D, Wang C, et al. CRTH2 promotes endoplasmic reticulum stress-induced cardiomyocyte apoptosis through m-calpain. EMBO Mol Med. 2018;10(3). DOI:10.15252/emmm.201708237 PMC5840549 29335338

[33] Hong D, Gao HC, Wang X, et al. Asymmetric dimethylarginine triggers macrophage apoptosis via the endoplasmic reticulum stress pathway. Mol Cell Biochem. 2015;398(1–2):31–38. 25209804 10.1007/s11010-014-2202-4

[34] Li C, Wei J, Li Y, et al. Transmembrane Protein 214 (TMEM214) mediates endoplasmic reticulum stress-induced caspase 4 enzyme activation and apoptosis. J Biol Chem. 2013;288:17908–17917. 23661706 10.1074/jbc.M113.458836 PMC3682588

[35] Su Q, Wang Y, Yang X, et al. Inhibition of endoplasmic reticulum stress apoptosis by estrogen protects human umbilical vein endothelial cells through the PI3 kinase-akt signaling pathway. J Cell Biochem. 2017;118(12):4568–4574. 28485890 10.1002/jcb.26120

